# Acute Bickerstaff Brainstem Encephalitis in a Middle-Aged Male: Diagnostic Challenges and Therapeutic Responses

**DOI:** 10.7759/cureus.87964

**Published:** 2025-07-15

**Authors:** Ameed Bawwab, Issa Snoubar, Orabi Hajjeh, Lana Khatib, Danny Aiti

**Affiliations:** 1 Department of Internal Medicine, Cleveland Clinic Mercy Hospital, Canton, USA; 2 Department of Internal Medicine, Cleveland Medical Center, University Hospitals, Cleveland, USA; 3 Department of Medicine, An-Najah National University, Nablus, PSE; 4 Department of Internal Medicine, Cleveland Clinic Fairview Hospital, Cleveland, USA; 5 Department of Internal Medicine, Canton Medical Education Foundation, Canton, USA

**Keywords:** anti-gq1b antibodies, autoimmune encephalitis, bickerstaff brainstem encephalitis, guillain-barré spectrum, ophthalmoplegia

## Abstract

Bickerstaff brainstem encephalitis (BBE) is a rare, post-infectious autoimmune disorder characterized by ophthalmoplegia, ataxia, and altered consciousness. It shares overlapping features with Guillain-Barré syndrome (GBS) and Miller Fisher syndrome (MFS), and diagnosis is often clinical, supported by anti-GQ1b antibodies. A 40-year-old previously healthy male presented with dizziness, diplopia, gait instability, and a recent viral prodrome. Neurologic examination revealed bilateral sixth cranial nerve (CN VI) palsy, partial involvement of the third cranial nerve (CN III), and bilateral dysmetria. Brain Imaging was unremarkable. Cerebrospinal fluid (CSF) analysis revealed lymphocytic pleocytosis, and the infectious workup was negative. Serum Anti-GQ1b antibody levels were elevated. The patient was treated with intravenous immunoglobulin (IVIG), resulting in marked improvement over four days; however, mild deficits persisted at the time of discharge to a rehabilitation facility. BBE remains a diagnostic challenge due to its rarity and nonspecific findings. Brain magnetic resonance imaging (MRI) may be normal, and anti-GQ1b antibodies, though not always present, are valuable for diagnosis. Prompt treatment with IVIG improves prognosis. This case exemplifies the clinical overlap within the Fisher-Bickerstaff spectrum and the importance of early recognition. Early identification of BBE is crucial for timely treatment and improved outcomes. This case underscores the need to consider autoimmune encephalitis in patients with acute brainstem symptoms and recent viral illness, even when imaging is normal.

## Introduction

Bickerstaff brainstem encephalitis (BBE) is a rare autoimmune neurological disorder characterized by autoantibody production against GQ1b ganglioside, which leads to inflammation that affects the brainstem [1]. It often follows upper respiratory or gastrointestinal infections [2].

BBE is characterized by a triad of ophthalmoplegia, ataxia, and disturbances in consciousness [3]. These disturbances may indicate involvement of the central nervous system (CNS), which distinguishes Bickerstaff encephalitis from Guillain-Barré syndrome (GBS) and Miller Fisher syndrome (MFS), as there is an overlap between these three diseases clinically and in the laboratory [3,4].

Diagnosing BBE is usually difficult; it relies on several features, including historical, clinical, and laboratory aspects such as the anti-GQ1b antibody test, and radiological aspects, including magnetic resonance imaging (MRI) and other modalities [5].

The treatment of BBE may include several options, like intravenous immunoglobulin (IVIG), plasma exchange, or corticosteroids [6]. Early detection of the disease leads to effective therapeutic intervention, which improves the state of the patient.

In this case, we present a 40-year-old male with acute dizziness, visual disturbances, and gait abnormality. We confirmed the diagnosis of BBE early with elevated serum anti-GQ1b antibodies. Then we administered IVIG, and we noticed significant improvement over four days.

## Case presentation

A 40-year-old healthy male with no significant past medical history presented with an acute onset of dizziness, visual disturbances, and gait abnormality. He reported a headache, neck stiffness, and vertical binocular diplopia, with a preceding viral prodrome a few days before his presentation. On physician examination, a neurological examination revealed bilateral cranial nerve (CN) 6 palsy, left CN 3 palsy, restricted extraocular movements, and bilateral dysmetria. Cardiovascular, respiratory, and abdominal exams were unremarkable. The patient was admitted for further evaluation and treated empirically with antibiotics for presumed meningitis. Consultations with infectious disease and neurology specialists were sought. An extensive diagnostic workup included Brain and spine imaging, CT head, CT angiography (CTA) head and neck, and MRI of the brain, which were all unremarkable. Lumbar puncture (LP) was performed, and cerebrospinal fluid (CSF) analysis showed white blood cells (WBC) 359/mm³ (80% lymphocytes), protein 47 mg/dL, and glucose 82 mg/dL. CSF microbiological tests were negative for Listeria, Streptococcus, Escherichia coli, Haemophilus influenzae, Herpes Simplex Virus 1 (HSV 1) and HSV 2, varicella zoster virus (VZV), Cryptococcus, Venereal Disease Research Laboratory (VDRL), enterovirus, West Nile virus (WNV), and Francisella tularensis antibodies. Human immunodeficiency virus (HIV) was non-reactive, and serum anti-GQ1b antibody levels were elevated at 192 IV (Table 1).

**Table 1:** Key laboratory and CSF findings. CSF, cerebrospinal fluid; PCR, polymerase chain reaction; IV, intravenous

Test	Patient value	Reference range	Interpretation
CSF white blood cells (WBC)	359/mm³	0-5/mm³	Elevated
CSF differential	80% lymphocytes	Predominantly lymphocytic	Suggesting for viral or autoimmune
CSF protein	47 mg/dL	15-45 mg/dL	Mildly elevated
CSF glucose	82 mg/dL	40-70 mg/dL	Mildly elevated
CSF bacterial cultures	Negative	Negative	No bacterial growth
CSF viral PCRs	Negative for HSV, VZV, enterovirus, WNV	Negative	No viral etiology identified
HIV	Non-reactive	Non-reactive	Negative
Anti-GQ1b Antibodies	192 IV	0-50 IV	Strong positive

Antibiotic therapy was discontinued given negative cultures and serologies. The patient was diagnosed with BBE based on the clinical presentation and elevated serum GQ1b antibody levels. Following the diagnosis, the patient was treated with a four-day course of IVIG, resulting in significant clinical improvement in ocular movement and gait instability but not complete resolution. The patient was discharged to an acute rehabilitation facility for physical and occupational therapy to address residual symptoms.

## Discussion

In 1951, Edwin Bickerstaff described three cases of altered mental status, ophthalmoplegia, and ataxia, naming them “mesencephalitis and rhombencephalitis.” Later, he expanded the study and termed it “brainstem encephalitis,” now known as BBE. BBE falls within the *Fisher-Bickerstaff syndrome* spectrum and shares features with GBS and MFS, with anti-GQ1b antibodies often present [3,4].

BBE is a rare autoimmune, demyelinating condition affecting the brainstem. It is often triggered by previous infections like Campylobacter jejuni, Mycoplasma pneumoniae, or H. influenzae. BBE cases were also reported after COVID-19, Listeria, Shingles, and aspergillosis, which further highlights previous infections as a trigger for the condition [7-10]. Molecular mimicry likely causes immune-mediated brainstem inflammation and demyelination.

The disease is rare, with unclear prevalence due to underreporting and diagnostic challenges. A national study conducted in Japan showed an estimated annual incidence of BBE of approximately 0.078 per 100,000 inhabitants with male predominance [11].

The clinical manifestations of the disease are varied between cases but mainly include progressive, symmetric external ophthalmoplegia and ataxia for four weeks, along with consciousness disturbance or hyperreflexia as a diagnostic criterion [5]. However, other clinical manifestations can be reported, such as headache, vomiting, fever, facial weakness, bulbar weakness, and limb weakness [12].

The diagnosis of BBE is often challenging. It is based on a combination of anamnestic, clinical, and radiological features [13]. Although there is no specific biological marker for BBE yet, serum antiganglioside antibodies (anti-GQ1b) could be positive in nearly two-thirds of cases [12]. Elevated levels could lead to earlier diagnosis and earlier intervention, leading to better outcomes, as in our case. However, the absence of this marker does not exclude BBE.

To rule out infectious meningitis, lumbar punctures are routinely carried out. According to CSF examination, 40% of cases have lymphocytic pleocytosis, 59% have hyperproteinorrachia, and 19% have albuminocytologic dissociation [14,15].

Electroencephalography (EEG) and electromyography (EMG) can aid in the diagnostic evaluation. In a review of 74 cases, EEG revealed abnormalities in over 85% of patients, most notably unarousable sleep-like patterns indicative of brainstem dysfunction, while EMG demonstrated features of demyelination or axonal injury in more than 55% of cases [12]. Imaging is also considered when dealing with BBE cases. CT scans could be used, although they will not show any abnormalities in BBE. On the other hand, the gold standard technique for brain imaging in encephalitis has been shown to reveal abnormalities in 41.4% of cases, most commonly hyperintensities in the brainstem (particularly the pons and midbrain), with occasional involvement of the cerebellum, thalamus, or cerebral cortex [12].

To date, there are no established guidelines for treatment. The mainstay of treatment is immunomodulation, including IVIG as first-line [6] and plasmapheresis. In some cases, the use of steroids could be combined with the main treatment [12]. Recently, there has been promising research showing the benefit of using rituximab in severe cases not responding to typical management [16].

Untreated BBE can cause severe complications, including permanent ataxia, ophthalmoplegia, and respiratory dysfunction. But in general, the disease has a good prognosis and recovery within four to six weeks after starting the treatment [17].

## Conclusions

This case highlights the diagnostic challenges and therapeutic response in BBE, a rare and often underrecognized condition. Despite an initial misdiagnosis and empiric antibiotic therapy for suspected meningitis, the diagnosis of BBE was confirmed through elevated anti-GQ1b antibodies, leading to successful treatment with IVIG. This case underscores the importance of considering rare autoimmune conditions in the differential diagnosis of neurological symptoms, particularly following a viral prodrome. Early recognition and prompt treatment with IVIG are crucial for favorable outcomes, although residual symptoms may persist in some cases. Enhanced awareness of BBE among clinicians can facilitate timely diagnosis and intervention, ultimately improving patient prognosis.
